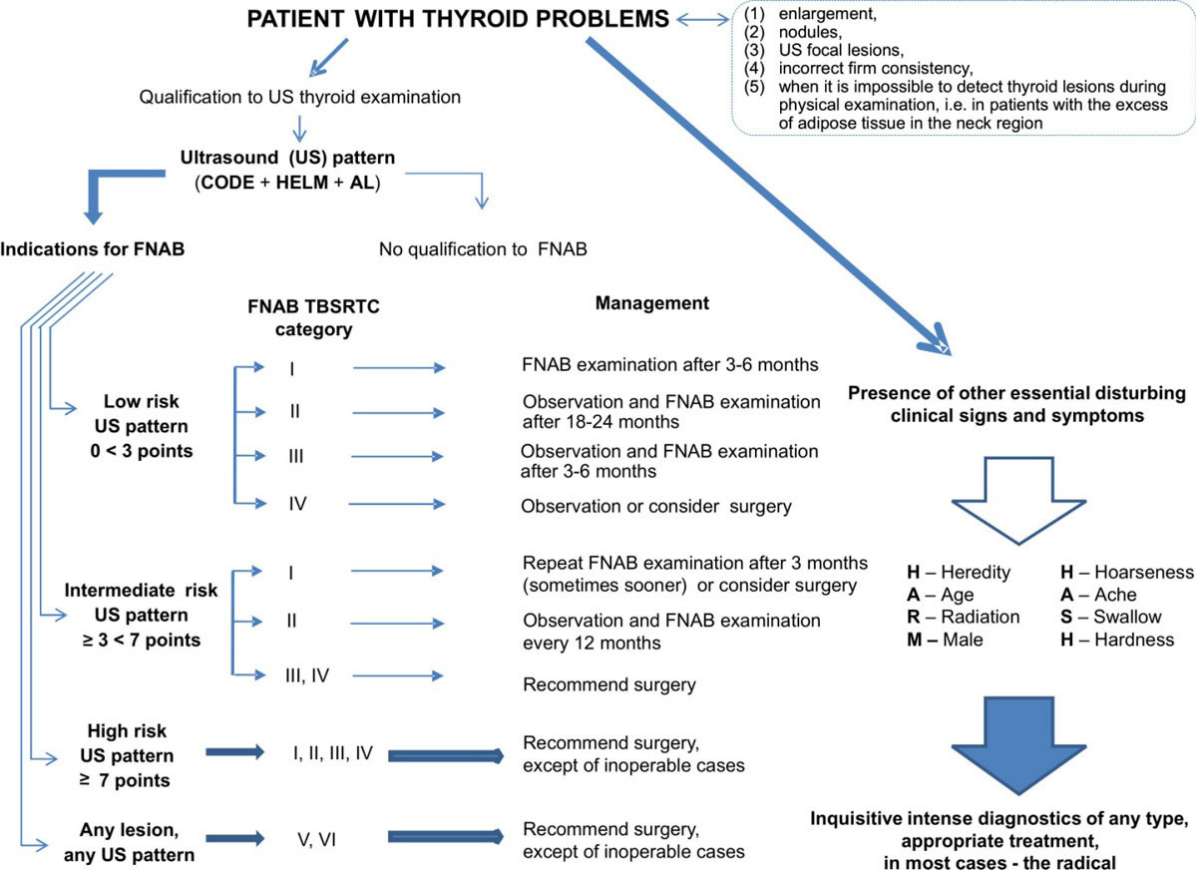# Ultrasound and cytological diagnostics of thyroid - its proper application in case of coexisting disturbing clinical signs and symptoms, suggestive of active proliferative lesion

**DOI:** 10.1186/1756-6614-8-S1-A19

**Published:** 2015-06-22

**Authors:** Andrzej Lewiński, Zbigniew Adamczewski

**Affiliations:** 1 Department of Endocrinology and Metabolic Diseases, Medical University of Lodz, Polish Mother’s Memorial Hospital – Research Institute, Lodz, Poland

## 

Currently, the most important clinical issue for a practicing endocrinologist is to answer the question whether the detection of a thyroid nodule/nodules during physical examination or thyroid focal lesion/lesions in the ultrasound (US) scan provides the basis for referring the patient for surgery.

The fine-needle aspiration biopsy (FNAB) performance is recommended for each case of:

1) a palpable nodule corresponding to focal lesion with diameter of 5 mm or more, revealed during US examination,

2) an impalpable lesion with a diameter of 10 mm and more,

3) a lesion with suspicious US features, suggesting the malignancy (see: Table 1).

**Table 1:** Definitions of suspicious US features (the, so-called, US patterns), in contrast to US patterns speaking for a benign nature of lesion [1].

Calcifications	- assessed especially as the presence of microcalcifications, also their coexistence with other forms of calcifications (e.g. dystrophic), in contrast to the absence of calcifications (the latter suggesting benign nature of lesions);
Orientation	- “taller-than-wide” shape on transverse and longitudinal planes, in contrast to all other shapes;

Doppler	- the presence of increased irregular chaotic central blood flows; this group includes also hypoechoic lesions when accompanied by a total absence of blood flow; in contrast to the peripheral, subcapsular blood flow (suggesting benign lesions);

Echogenicity	- hypoechogenicity, defined as “darker” than normal thyroid echogenicity, and described as similar to the echogenicity of muscles surrounding the gland, especially sternocleidomastoid muscles;

Halo	- uneven thickness of halo (outer shell that surrounds the lesion) or absence of halo, in contrast to thin halo, regularly surrounding the lesion;

Echostructure (composition)	- solid lesions, also mixed lesions with cystic portion not exceeding 10% of the volume, in contrast to mixed lesions with cystic parts greater than 10% of total volume, as well as to lesions with solely cystic composition;

Largeness (size)	- the size of lesion greater than 3 cm in diameter, in contrast to smaller lesions;

Margin	- poorly defined and irregular, sometimes infiltrated border, in contrast to a well-differentiated regular margin;

Augmentation	- the enlargement of lesion by at least 20% in two dimensions, i.e. at least 50% by volume (for the lesion in diameter of less than 10 mm - minimum 2 mm in two dimensions) in a period of time shorter than 1.5 years [2,3];

Lymph node invasion	- the presence of lymph nodes, suspicious in US evaluation and of the size larger than 5-8 mm in the smallest dimension [3].

At the present stage, US examination still cannot be conducted with such precision so that it alone will be able to document the occurrence of malignancy and constitute a definitive diagnosis of thyroid cancer. Simultaneous visualization of even a few suspicious US features, as well as demonstration of enlarged cervical lymph nodes, together with observations of changes with time of thyroid lesions and of lymph nodes, can only bring us closer to a final diagnosis.

The most suspicious feature is the shape of a nodule/US focal lesion. Accordingly, "standing egg” or “taller-than-wide” shape - on transverse or longitudinal planes - can much more likely be related to the presence of malignancy [4]. This feature is estimated to be present in approximately 90% malignant cases [5]. This is due to the fact that malignant lesions grow across the normal tissue plane in a centrifugal manner [6].

We have grouped the individual US features into mnemonics, to facilitate their permanent memorising. Mnemonics are made up of the first letters of the names of these characteristics. Our experience allows us to propose a system that is based on assigning the points to each US feature. The principle of assessment is to add points, which will allow classification of the lesions to particular groups of a different risk of malignancy (Table 2).

**Table 2:** The scoring system of US features (patterns) assessed in thyroid nodules/focal lesions. Low risk US pattern – 0 < 3 points; intermediate risk US pattern – ³ 3 < 7 points; high risk US pattern - ³ 7 points (note scoring system modification when compared with ref. [1]).

**CODE** (each feature – 1 point)		
C	Calcifications	Max. no. of points – 4
O	Orientation	
D	Doppler	
E	Echogenicity	

**HELM** (each feature – 0.5 point)		

H	Halo	Max. no. of points – 2
E	Echostructure	
L	Largeness	
M	Margin	

**AL** (each feature – 3 points)		

A	Augmentation	Max. no. of points – 6
L	Lymph node involvement	

Two signs/symptoms presented in the bottom of Table 2 are of crucial significance since they indicate a serious suspicion of the malignancy. Thus, occurrence of each of these two features is associated with granting of 3 points.

In addition to US data, one should account several clinical signs and symptoms, such as occurrence of hoarseness, dysphagia or pain which result from the presence of the thyroid tumour of firm consistency. Also of importance is information on inherited diseases, with particular stress on multiple endocrine neoplasia 2A and 2B (MEN 2A and 2B), familial medullary thyroid carcinoma (FMTC) or familial non-MTC (FNMTC) in 1st degree relatives, as well as history of neck, head, chest or whole body irradiation - especially if it relates to the childhood. Another high risk factor of thyroid cancer that absolutely qualifies for further diagnostic investigation is patients’ age - under 20 and over 60 years, as well as male sex is regarded as a risk factor for malignancy of thyroid lesions.

The coexistence of essential disturbing clinical signs and symptoms, mentioned above, provide additional data indicating an increased risk of the malignant neoplastic disease. For easier memorising the list of disturbing signs and symptoms, we propose two subsequent mnemonics illustrated in Table 3.

**Table 3:** Disturbing signs and symptoms that require inquisitive intense diagnostics, regardless of The Bethesda System for Reporting Thyroid Cytopathology (TBSRTC) category and US pattern [1].

HARM		HASH	
H	Heredity	H	Hoarseness
A	Age	A	Ache
R	Radiation	S	Swallow
M	Male	H	Hardness

According to our opinion, introduction of the scoring system for the disturbing symptoms and signs is useless because their assessment can be simply achieved by applying common sense. In other words, the occurrence of almost all of these symptoms by itself requires the execution of all possible diagnostic tests, followed by treatment implementation. In any case, a reassuring outcome of US and FNAB examination should not cause failure to the appropriate diagnostics and treatment.

Most authors assume that FNAB of the thyroid is a basic procedure for qualifying the patient for surgery or clinical observation. Generally accepted classification of FNAB diagnostic categories is shown in Table 4[7].

**Table 4:** The Bethesda System for Reporting Thyroid Cytopathology (TBSRTC) – according to Cibas and Ali [7].

Diagnostic category		Risk of malignancy (%)
I II III IV V VI	Nondiagnostic or unsatisfactory Benign Atypia of undetermined significance or follicular lesion of undetermined significance Follicular neoplasm or suspicious for a follicular neoplasm Suspicious for malignancy Malignant	1-4 0-3 5-15 15-30 60-75 97-99

However, one must be aware of aspiration cytology limitations, resulting from the specificity of FNAB technique itself. This entails the risk of false negative results of a few percent, and less - false positives results. Even a diagnosis of "benign lesion" (category II acc. TBSRTC), assuming a proper sample collection from the tested lesion, is still associated with a risk of cancer but less than 3% [7]. Nevertheless, the authors of recent study suggest that repeated FNAB in the category in question can be performed only after 2 to 4 years, if the first FNAB indicated benign character of the lesion; the above recommendation applies to lesions in asymptomatic patients with no risk factors [8].

FNAB limitations result also from the nature of an assessed lesion itself, especially in situations when the final diagnosis can be formulated only on the basis of histopathological examination - e.g. follicular thyroid carcinoma (FTC). Diagnoses of "follicular lesion of undetermined significance (FLUS)/atypia of undetermined significance (AUS)" (category III TBSRTC) [6] or of "follicular-neoplasm/suspicious for follicular neoplasm" (category IV) relate - in most cases - to hyperplastic nodules or follicular adenoma rather than to FTC. In practice, it means that follicular adenoma and FTC cannot be distinguished on the basis of cytological examination.

According to TBSRTC recommendations [^7^], category III should only be used in exceptional situations when it is not possible to establish a more precise cytological diagnosis since cytological findings are not convincingly benign to be classified as category II, yet the degree of cellular or architectural changes is not sufficient for an interpretation of category IV and - even more - of category V (“suspicious for malignancy”). Moreover, the authors of recent study suggest that the patients with cytological diagnosis - AUS (within category III TBSRTC) should be qualified to surgery much more often than the patients with FLUS (also category III), because of significantly higher risk of malignancy [9].

Not every FNAB result may precisely identify what type of treatment should be applied; all non-diagnostic or unsatisfactory smears are assigned to category I. Such a result is an indication for repeat biopsy, usually within 3-6 months, sometimes sooner, because the risk of malignancy is not quite small and it is rated for a few percent.

The most important objective of our presentation is to propose an algorithm of diagnostic and therapeutic management in thyroid nodules/US focal lesions which is based on the information from both US image and FNAB cytology. In Figure 1 we have presented the modified version of our earlier algorithms [1,4]; for modification we have taken into account the recent reports of other authors [8,9].

**Figure 1:**